# Botrops derived hemocoagulase formulation a probable agent for diabetic wound healing

**DOI:** 10.1007/s13205-020-02429-5

**Published:** 2020-09-18

**Authors:** Raghuvir Keni, Karthik Gourishetti, Manas Kinra, Pawan G. Nayak, Rekha Shenoy, Krishnadas Nandakumar, Rajesh N. Jagdale, K. V. Raghavendra, Syed Mushtaq Ahmed

**Affiliations:** 1 Department of Pharmacology, Manipal College of Pharmaceutical Sciences, Manipal Academy of Higher Education, Manipal, 576104 Karnataka India; 2 Juggat Pharma, Jagdale Industries Pvt. Ltd., Bengaluru, 560078 Karnataka India

**Keywords:** Hemocoagulase, *Botrops atrox*, Venom, Diabetic wound

## Abstract

Botroclot is a marketed preparation containing hemocoagulase, which is an enzyme having coagulant activity, isolated from the snake *Botrops atrox.* This formulation is used in dental surgeries and other minor surgical wounds. However, the formulation remains untested in diabetic wounds. Hence, we proposed a study for the topical application of Botroclot in high-fat diet (HFD) + Streptozotocin (STZ) induced diabetic rats. HFD was fed initially to rats which facilitates the development of insulin resistance. Thereafter, an injection of STZ (40 mg/kg, i.p.) was given. This resulted in the development of diabetes with elevated fasting glucose and impaired glucose tolerance. After stabilization of blood glucose values, wounds were created by punch biopsy on the dorsal side of the palm of the rat to mimic the diabetic wounds frequently seen in the case of humans. Later, the application of Botroclot on these wounds was carried out for 15 days. Topical application of hemocoagulase improved the wound closure and there was a gradual decrease in inflammatory markers and a substantial increase in collagen deposition occurred. Histopathological findings indicated the same, with an increase in granulation tissue suggesting that the topical application moderately improves the wound healing in diabetic rats. We conclude that Botroclot can have a mild to moderate effect in improving collagen deposition and thus wound contraction, improving wound closure in diabetic wounds in rats. This study also establishes the basis for exploration of agents from venom-based sources in diabetic wound healing.

## Introduction

Diabetic wounds are a common issue seen with patients. As compared to wound healing in healthy individuals, healing of the wound is delayed in case of diabetic patients. Physiologically the reason is due to impaired blood flow, diabetic neuropathy and impaired immune function. Whereas at a molecular level, the impaired cytokine production, impaired fibroblast and other cell migration and chronic inflammation are associated with the delay in wound healing (Patel et al. 2019). Diabetic wound healing and diabetic ulcers remain an unaddressed area with no agents other than Becaplermin (Steed 1995) available to hasten the healing of wounds, however even this agent has now been withdrawn due to the risk of cancer at the site of application as well as at distant sites (EMA 2012). Diabetic wounds are afflicted by increased infection, inflammation and delayed or insufficient collagen deposition that delays wound healing (Fahey et al. 1991). Plant-derived product is an avenue that has been tried for all kinds of wounds and conditions, whereas natural products of animal origin is an area that is still in its infancy. Venoms are a mixture of compounds from animal sources used for defence and hunting prey which can be investigated for the purpose of wound healing (Estevão-Costa et al. 2018), with bee venom being one agent that has been previously investigated for this purpose (Badr et al. 2016).

Botroclot is a marketed topical solution containing sterile hemocoagulase solution 0.2 CU, isolated from *Bothrops atrox* or *Bothrops jararaca* venom along with chlorhexidine (0.1%v/v). Hemocoagulase is an enzyme usually found in venoms of hemotoxic snakes that catalyse the coagulation of blood. Its activity is associated with a molecule called Batroxobin (BTX) (Madhu et al. 2019). Over the years, many components of animal venom have been investigated for their potential use for a variety of diseases and disorders. Amongst these BTX is one such component that has been successfully tested and marketed for use as a topical coagulant for minor (dental) and major surgeries (Chowta et al. 2014; Madhu et al. 2019).

Hemocoagulase works by causing cleavage of fibrinogen into fibrin monomers which eventually leads to the formation of fibrin polymers (Chowta et al. 2014). The nature of BTX is characteristically different from that of thrombin and so are their resulting clots. BTX shows activity systemically as well as at the local site of application in the absence of important clotting factors. Thrombin is inactivated in the presence of antithrombin, which is not seen with BTX.

BTX has been shown to promote wound healing in dental surgeries (Vandana Shenoy et al. 2015), by improving collagen deposition, reducing inflammation and infection. In wounds of primary intention, BTX allows for a good seal between the edges and reduces scarring. In this paper, we investigate the role of Botroclot on diabetic wound healing in HFD-STZ diabetic rats an area in which this agent has not been tried for.

In streptozotocin-induced diabetic models, it has been observed that delayed wound healing is due to the impaired inflammatory phase. In this study, we have focused on the effect of Botroclot on various wound markers to investigate its potential for diabetic wounds.

## Materials and methods

### Materials

Streptozotocin (STZ) (MP Biomedicals India Pvt. Ltd., Navi Mumbai, India), Normal saline, ketamine and xylazine were purchased from a local pharmacy. Acrylamide, BCA protein assay kit and lysis buffer were procured from Thermo Fisher Scientific Inc., Illinois, USA. Formalin and sodium citrate used were of analytical grade. Non-fatty blocking grade milk ( Bio-Rad Laboratories, California, USA) bovine serum albumin (HiMedia Laboratories, Mumbai, India), antibodies (Elabscience Inc., Wuhan, China), enhanced chemiluminescent (ECL) reagent ( Thermo Fisher Scientific Inc., Illinois, USA) were also procured.

## Methods

Animal care and handling were done with the approval of Committee for the Purpose of Control and Supervision of Experiments on Animals (CPCSEA) study approval number IAEC/KMC/114/2018. Healthy inbred male albino rats of Wistar strain (180–220 g) were used for the wound models. They were maintained under controlled conditions of temperature (23 ± 2 °C), humidity (50 ± 55%) and day/night cycles (14 h light and 10 h dark) at Central Animal Research Facility, Manipal. The animals were provided with food and water ad libitum. Each animal was housed in a separate polypropylene cage containing paddy husk as bedding.

### Induction of diabetes

Diabetes was induced in rats by feeding a high-fat diet (HDF) followed by STZ at a dose of 40 mg/kg i.p. (Reed et al. 2000; Srinivasan et al. 2005). In short, rats were divided into two groups normal control which was fed normal chow and the diabetic group which was provided HFD. Rats were first fed with HFD containing 50% fat in the form of lard as the source of calories for 8 weeks. The composition of the feed used in the study was described as mentioned elsewhere (Srinivasan et al. 2004, 2005). Rats that showed impaired oral glucose tolerance test (OGTT), conducted with the help of handheld glucometer (Alere G1 AGM-4000; Alere Medical Pvt. Ltd., Haryana, India), were considered for further treatment and were placed in the diabetic group. Animals from the diabetic group were then fasted for at least 8 h and injected with 40 mg/kg of STZ dissolved in citrate buffer having pH 4.4 for induction of diabetes. After 4 weeks of STZ injection, those animals which showed fasting blood glucose levels greater than 400 mg/dL were selected for further study. Animals were then grouped as normal control, diabetic control, and treatment with each group having 6 animals and weight recorded at 3, 6 and 8 weeks.

### Creation of wound and treatment

After the stabilization period, rats were anesthetized using ketamine and xylazine (Ketamine 60 mg/kg and xylazine at 10 mg/kg both given by intraperitoneal route), and a full-thickness wound of 6 mm diameter (excision model) was made using punch biopsy (Ribbels dermal biopsy punch) (Trujillo et al. 2015; Yu et al. 2017) on the dorsal side of the palm of the rat. The treatment group of rats were applied Botroclot solution (1–2 drops daily) on the wound with the help of the applicator inbuilt into the bottle until the day of sacrifice. Images of wounds were captured using a digital camera on alternate days. No postoperative analgesia was provided after wounding.

Rats in normal control, disease control and treatment group were sacrificed at days 5, 10 and 15. The wound tissue was carefully cut and harvested and preserved either in formalin or lysis buffer for tissue histology and western blot, respectively. The tissue in lysis buffer with protease and phosphatase inhibitors was stored in liquid nitrogen until further processing.

### Haematoxylin and eosin staining

The tissue samples were stored in 10% formalin for fixation. Alcohol and xylene were used to dehydrate and clear the tissue after which it was put into wax moulds. Blocking or embedding the tissue was done to transfer the tissue from the final wax bath to a mould filled with molten paraffin wax. Thin sections of tissue blocks of 4 microns thickness were cut with the help of microtome. The tissue sections were floated in a water bath of temperature 50–52 °C and then taken in microscopic slides. After de-paraffinization with xylene and alcohol, the tissue was stained.

### Western blotting

The tissue was allowed to thaw and was maintained on ice throughout the process, after removal from liquid nitrogen. Tissue was homogenised with the help of a hand-held homogenizer (D1000; Benchmark Scientific, New Jersey, USA). The lysate solution was then centrifuged at 10,000 RPM at 4 °C using a cooling centrifuge (Mikro 22R; Andreas Hettich GmbH & Co. KG, Tuttlingen, Germany) and the supernatant collected.

Total protein quantification was done using BCA protein assay kit (Smith et al. 1985), 25 µg of total protein were loaded on to the gel. After the run process, the protein was transferred to a PVDF membrane and blocked with blocking grade nonfatty milk for 1 h and then washed with tris-buffered saline with 0.1% Tween 20 (TBST). The membranes were then incubated with primary antibodies for the following markers- tubulin, collagen 1, collagen 3 and Interleukin-6 individually overnight diluted with 5% bovine serum albumin.

The membranes were then washed with tris buffered saline with 0.1% Tween 20 and then incubated with secondary horseradish peroxidase labelled antibody for 1 h, diluted with 5% non-fatty milk. The blots were then washed again with TBST and developed with the help of imaging system (G:BOX Chemi XRQ; Syngene, Cambridge, UK) using enhanced chemiluminescence (ECL) reagent.

### Analysis

Wound areas were calculated using ImageJ software using photographs captured of the wounds. Percentage change in wound area was done based on the following formula:

$$\% \,\,{\text{of}} \, {\text{wound}} \,{\text{closure}} = \left( {\frac{{{\text{Initial day area}} {-} n{{{\text{th}}}} \, {\text{day}} \,{\text{area}}}}{{{\text{Initial}} \,{\text{day}} \,{\text{area}}}}} \right) \times 100$$

Areas were then analysed using ANOVA and Dunnett's post hoc test. Blots were analysed using ImageJ software.

## Results

### Wound area contraction

**Fig. 1:**
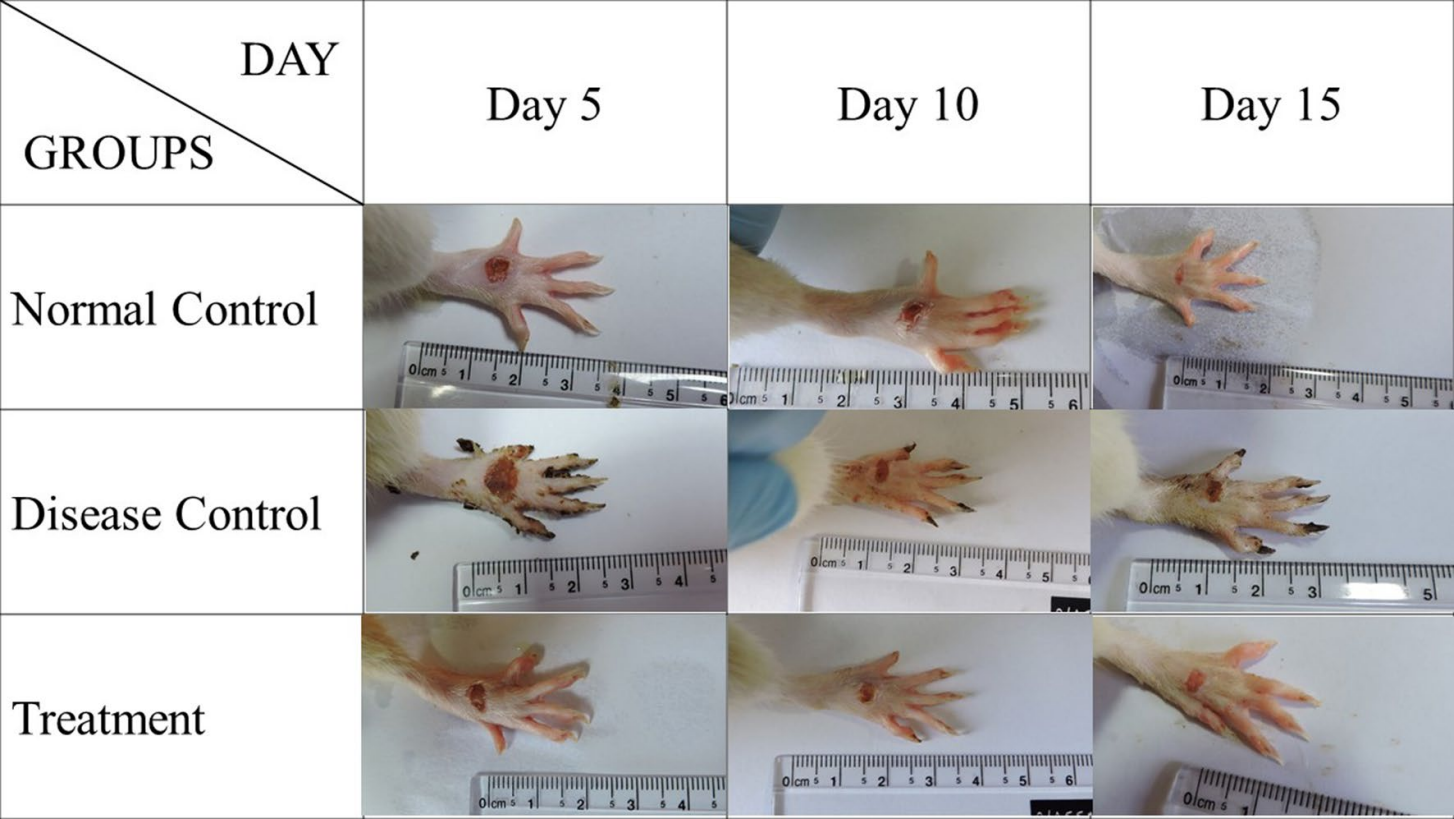
This figure displays the day-wise transition of the wound. At day 5, the wound of the disease control animal and botroclot treatment wound appears to be still fresh, by day 10 the disease control wound has still not formed a clear scab, but the treatment group wound has formed one. By day 15, the normal control wound is fully healed, and treatment group wound has also healed but the disease control has not yet fully healed

**Fig. 2:**
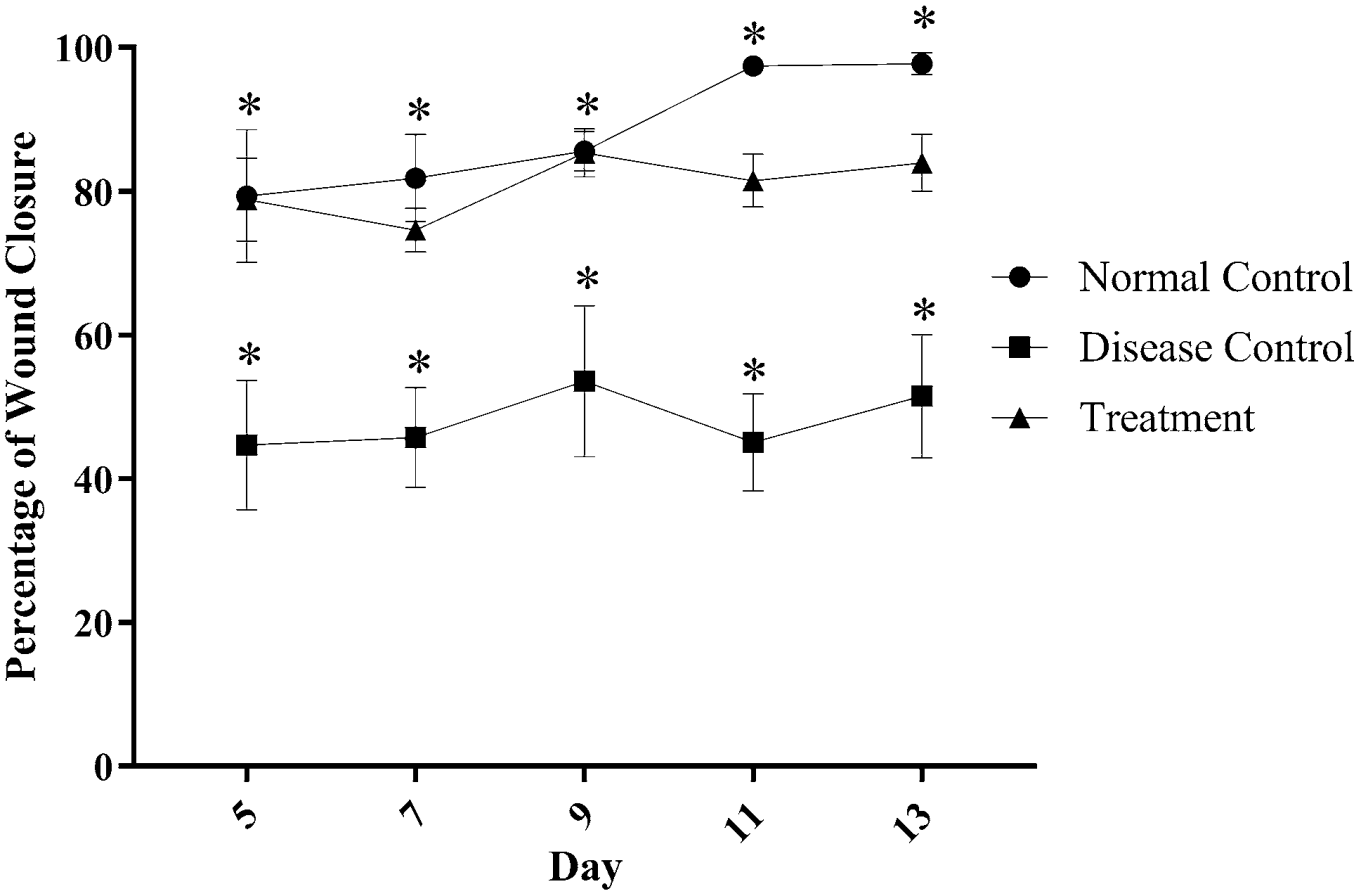
Wounds treated with Botroclot healed better than disease control wounds which barely achieved 50% closure. Data was found to be significant at *P < 0.05 when normal control and treatment groups vs. disease control using one-way ANOVA followed by Dunnett’s post-hoc test

The wound treated with Botroclot displayed a better reduction in the wound area and good shrinkage as compared to untreated wounds of the disease control group as seen in Figs. 1, 2. On day 5, the disease control group had only 40% wound closure, whereas treatment and normal control groups had 80% wound closure. By day 10, the disease control groups displayed only a slight increase in wound closure which did not improve further even up to day 13. On the other hand, the treatment group displayed a slight increase beyond 80% and normal control achieved a wound closure of up to 100%. Data was found to be significant at *P* < 0.05 in normal control and treatment groups vs. disease control using one-way ANOVA followed by Dunnett’s post-hoc test.

### Wound histopathology

**Fig. 3:**
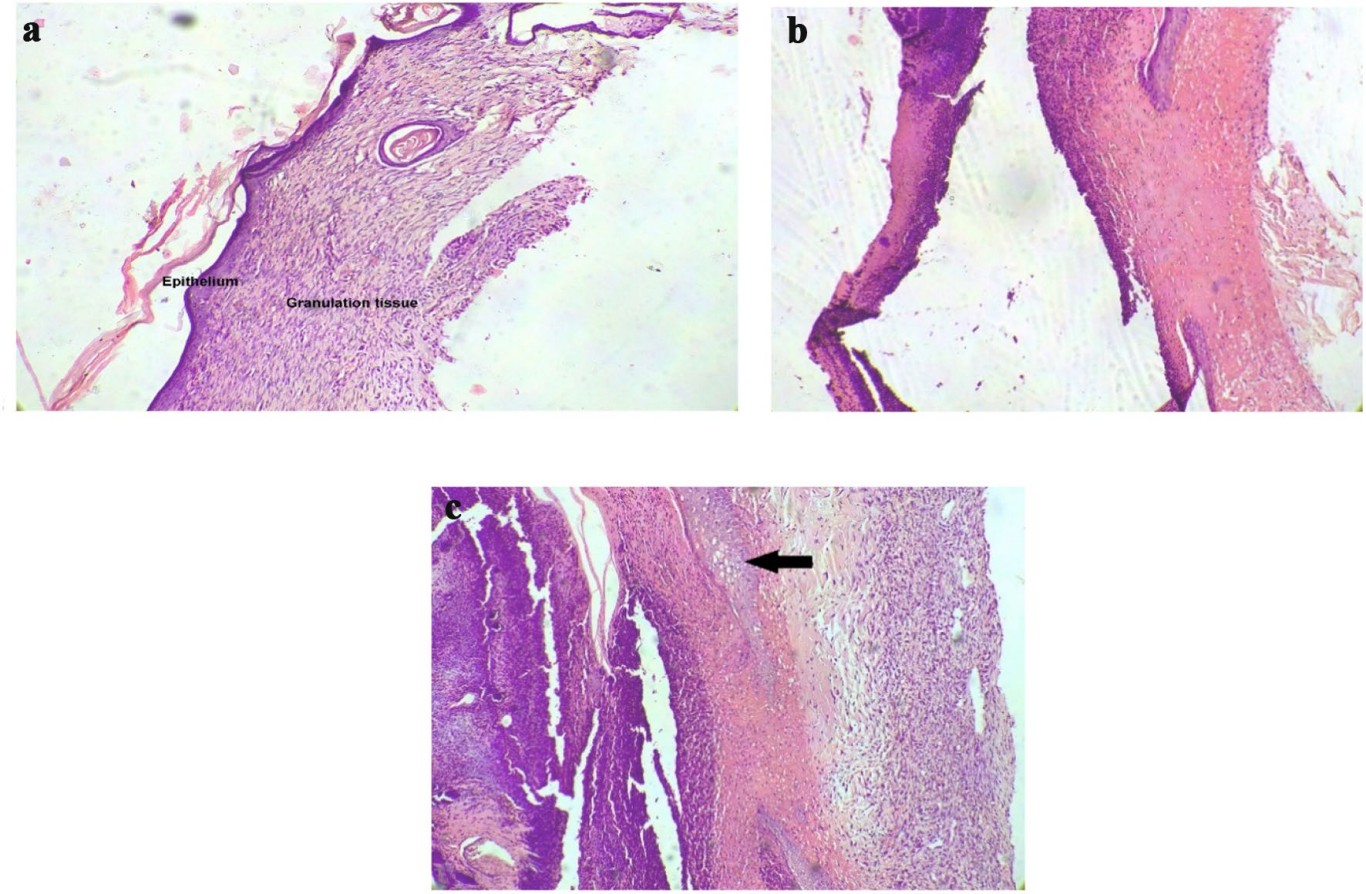
Histopathology slide images of wound taken at ×10 magnification on day 15 **a** Normal control wound section. a well-developed epithelial layer with underlying granulation tissue is seen indicating a well healing wound. **b** Disease control wound shows no granulation tissue formation **c** Botroclot treated wound section showing initiation of granulation tissue deposition

Through haematoxylin and eosin-stained sections, we wish to study collagen deposition, revascularization, and presence of new epithelial cells. We noticed the presence of ulcerative tissue in the disease control and treatment group. The normal control wounds showed the presence of regeneration early in the timeline unlike the other groups. Wounds treated with Botroclot showed signs of tissue regeneration and the presence of granulation tissue earlier as compared to disease control, despite the presence of inflammatory cells as seen in Fig. 3 and Table 1. The assessment was done using a histopathological scoring system as demonstrated by previous studies, with the help of a pathologist (Dogan et al. 2009; Gupta and Kumar 2015; Jamadagni et al. 2016).

**Table 1:** Histopathological changes observed by haematoxylin staining of wound sections

Group	Histopathological changes observed
Normal control	Tissue sections showed good epithelial regeneration and granulation tissue formation. The abundance of proliferating blood vessels and fibroblasts were seen with collagen formation
Disease control	The tissue sections showed ulcerated areas with mild epidermal regeneration. No granulation tissue formation is seen. The dermis is edematous with inflammatory cells
Treated	Tissue sections showed epidermal regeneration. Section showed good granulation tissue with proliferating blood vessels and fibroblasts and collagen formation

### Collagen deposition and inflammatory markers

Collagen I expression showed a gradual decrease from day 5 to 15 in the normal control groups but in case of disease control and treatment group no major variation was seen. An increase of collagen III was seen in all groups, in case of treatment group this increase was seen from day 5 onwards itself which was more than disease control.

A gradual increase in the levels of interleukin-6 was observed; the increase in case of Botroclot treated animals was more as compared to disease control and normal animals especially on day 15.

**Fig. 4:**
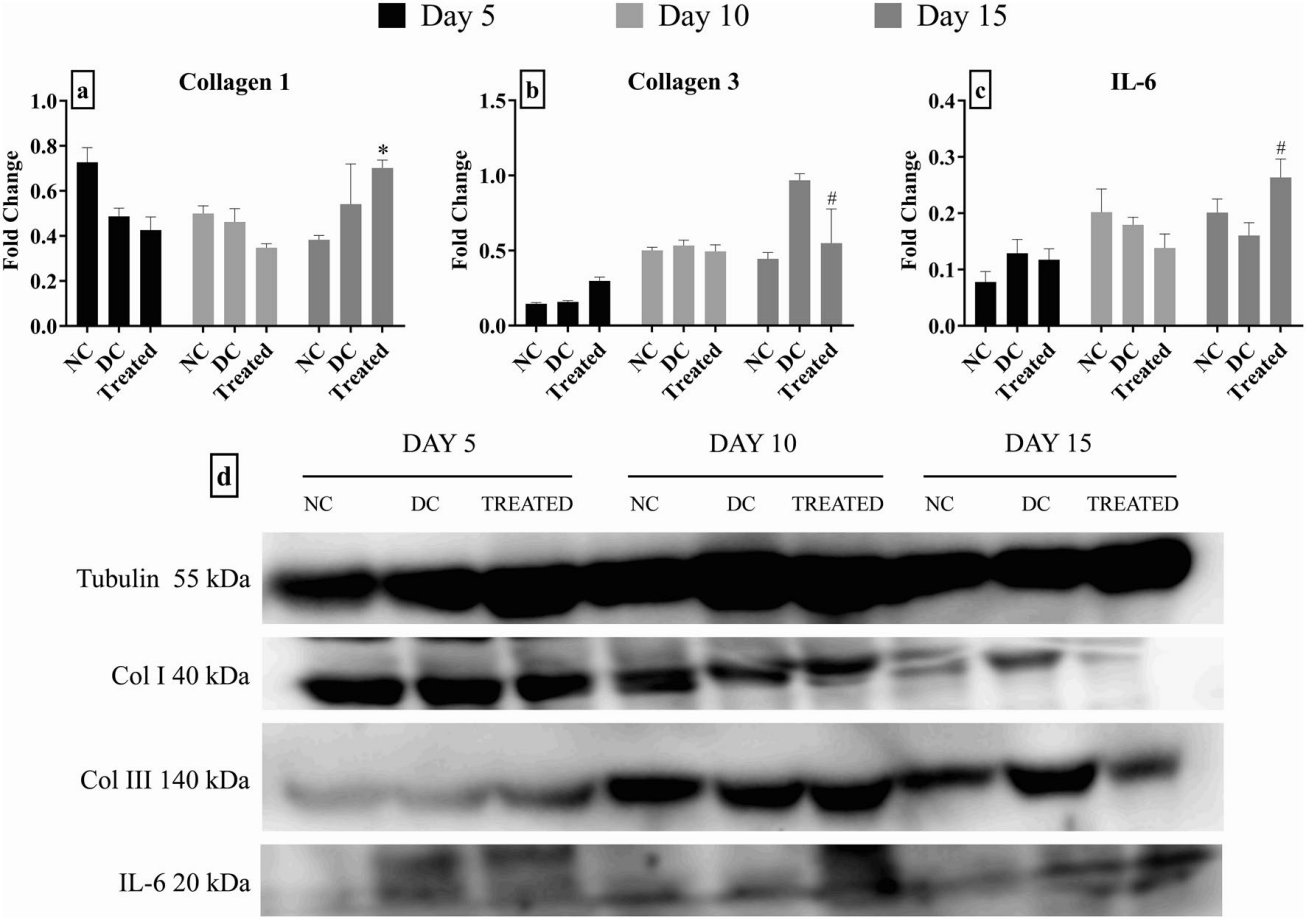
Effect of Botroclot on **a** Collagen 1—Treated group showed significant effect at *P < 0.01 when compared to normal control on day 15; **b** Collagen 3—Treated group was found to be significant at day 15 at ^#^*P* < 0.01 when compared to disease control; **c** IL-6—Treated group was found to be significant at day 15 at ^#^*P* < 0.05 when compared to disease control; **d** a representative image of the western blot taken with Syngene G:Box

Data was found to be statistically significant at day 15 as displayed in Fig. 4.

## Discussion

Botroclot in this study showed beneficial effects in hastening wound healing in diabetic rats as observed with shrinkage of the wound. Wound shrinkage occurs when there is migration of fibroblasts to the epithelial tissue that attaches into the matrix. The fibroblasts enter into the wound tissue and pull together the wound allowing for sealing/closure of the wound (Ehrlich and Hunt 2012; Brauer et al. 2019). This phenomenon helps restore the mechanical strength within the wound region. This process can only occur after or with the deposition of extracellular matrix in the form of collagen (Aravindan Rangaraj et al. 2011; Brauer et al. 2019). The deposition of ECM and collagen allows for the tissue cells to develop attachments and undergo modification and thus become permanent residents in the healing wound region. Through western blot and histopathological examination, we confirmed the promotion of collagen deposition and early granulation at the wound site. Type I and Type III are the major forms of collagen found in the skin (Clore et al. 1979). During healing, type III is initially deposited in the wound which is eventually converted into type I. Collagen deposition allows for keratinocyte and fibroblast migration (Guo et al. 1990; Aravindan Rangaraj et al. 2011; Lü et al. 2013; Jiang et al. 2016). It is for this reason that externally applied collagen provides an alternative form of the wound dressing. However, Botroclot provides a benefit here as it can be applied directly to a freshly formed wound and may prevent the conversion of the acute wound to a chronic wound, the collagen too is naturally generated at the wound and eliminates the external application of collagen. This observation is backed by histopathological examination of the tissue which has indicated the presence of granulation along with regeneration in wounds treated with Botroclot whereas, disease control wounds showed no deposition of granulation tissue but rather only mild regeneration indicating delayed healing.

The expression of pro-inflammatory marker IL-6 was found to be increasing towards day 15 which is similar to that of the normal healing process as seen from the western blot analysis. Even though Botroclot treated wounds healed faster, some amount of ulcerative tissue was seen in the case of both disease control and treated wounds in histopathological studies. In the case of diabetic wounds, inflammation is exacerbated which hinders the natural process of healing (Zhao et al. 2016). In diabetic rats, IL-6 is low in the early phase of wound healing, Botroclot does not cause a further reduction in this phase. Inflammatory markers tend to be elevated in the wound, attracting and allowing entry of immune cells. This, in turn, helps to clear debris and invading pathogens from the wound site only after which tissue repair can occur. Simultaneously, cytokines are released to counterbalance this effect (Acosta et al. 2008). If the inflammation does not subside, the wound gets converted into a non-healing wound. This is manifested in a diabetic wound where the counterbalance is not achieved. Non-healing wound needs to be debrided and ulcerative tissue to be excised to allow normal healing by secondary intention. Our findings suggest that Botroclot can provide some benefit in case of diabetic wound by improved deposition of collagen and thus wound closure, besides from the obvious early clot formation but not by reduction of inflammation.

The wound was measured only in two dimensions, measurement of paw volume can be a better tool for measuring healing in a three-dimensional manner.

We conclude that Botroclot can improve the rate of diabetic wound healing, as well as cause a reduction in the inflammation of wounds. It improves collagen deposition and shrinkage of the wound, thus causing faster closure as compared to non-treated wound closely simulating a normal non-diabetic wound in rats.

The effect of Botroclot in the reduction of infection and bacterial load on the wound, owing to the clotting property of the enzyme was not studied since it was beyond the scope of this study. Due to improved clot formation, the clot can provide a barrier to the entry of microbes. This can further improve healing.

## Conclusion

Botroclot appears to have a mild to moderate effect in diabetic wound healing in rats. This effect can be studied further to tailor the drug to ensure a more robust and potent effect on diabetic wound. Since it can hasten the effect of healing and is not a growth factor-like agent, it is safer as compared to other agents being tested. It does not affect the early phases of wound healing; however, it has displayed beneficial effects in the late phases of wound healing.
